# Logic-gated and contextual control of immunotherapy for solid tumors: contrasting multi-specific T cell engagers and CAR-T cell therapies

**DOI:** 10.3389/fimmu.2024.1490911

**Published:** 2024-11-13

**Authors:** Olivier Nolan-Stevaux, Richard Smith

**Affiliations:** ^1^ Oncology Research, Gilead Sciences, Foster City, CA, United States; ^2^ Cell Biology Research, Kite Pharma, Foster City, CA, United States

**Keywords:** T cell bispecific antibodies, CAR-T, logic gate, OR gate, AND gate, NOT gate, multispecific, T cell engagers

## Abstract

CAR-T cell and T cell engager therapies have demonstrated transformational efficacy against hematological malignancies, but achieving efficacy in solid tumors has been more challenging, in large part because of on-target/off-tumor toxicities and sub-optimal T cell anti-tumor cytotoxic functions. Here, we discuss engineering solutions that exploit biological properties of solid tumors to overcome these challenges. Using logic gates as a framework, we categorize the numerous approaches that leverage two inputs instead of one to achieve better cancer selectivity or efficacy in solid tumors with dual-input CAR-Ts or multi-specific TCEs. In addition to the “OR gate” and “AND gate” approaches that leverage dual tumor antigen targeting, we also review “contextual AND gate” technologies whereby continuous cancer-selective inputs such a pH, hypoxia, target density, tumor proteases, and immune-suppressive cytokine gradients can be creatively incorporated in therapy designs. We also introduce the notion of “output directionality” to distinguish dual-input strategies that mechanistically impact cancer cell killing or T cell fitness. Finally, we contrast the feasibility and potential benefits of the various approaches using CAR-T and TCE therapeutics and discuss why the promising “IF/THEN” and “NOT” gate types pertain more specifically to CAR-T therapies, but can also succeed by integrating both technologies.

## 1 T cell engagers and CAR-T therapies: successes, challenges, and opportunities

T cell Engagers bispecific antibodies (TCEs) and chimeric antigen receptor T cells (CAR-T) with transformative therapeutic effects have been approved for the treatment of Acute Lymphoblastic Leukemia (B-ALL) (1, 2), Non-Hodgkin’s Lymphoma (NHL) (3, 4), and Multiple Myeloma (MM) (5, 6), demonstrating the remarkable efficacy of these classes of therapeutics against advanced disseminated hematological malignancies, and in patients who relapsed after multiple lines of therapy. The sensitivity of B cell lineage malignancies (B-ALL, NHL, and MM) to TCEs and CAR-Ts is even more remarkable since these cancer types are typically poorly infiltrated by T cells and refractory to Immune Checkpoint Blockade (ICB) therapy (7–9).

**Figure 1:**
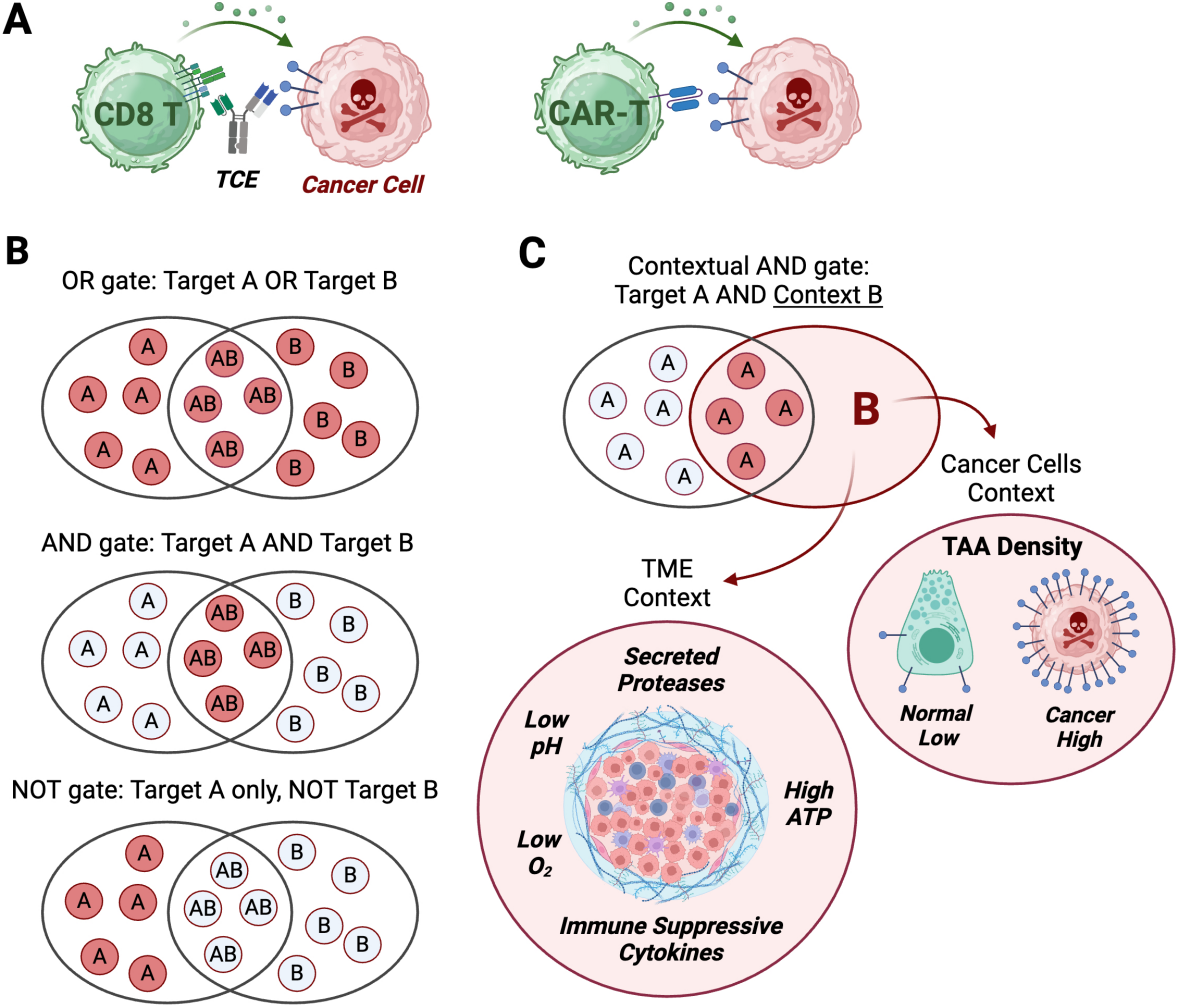
Logic gates applied to TCE and CAR-T modalities. **(A)** T cell engagers (TCE) and chimeric antigen receptor T cells (CAR-T) turn an input (target binding on cancer cells) into an output: cancer cell killing. **(B)** Three types of logic gates integrate two possible binding events (dual input) into a binary output: kill (red discs)/do not kill (white discs). OR gate logic leads to a cytotoxic output if target A, target B or target A and B are detected on cancer cells. AND gate logic induces a cytotoxic output only if target A and target B are detected on cancer cells. NOT gate logic triggers a cytotoxic output only if target A is detected, while target B is not detected on cancer cells. **(C)** Contextual AND gate is a logic gate integrating a single binding input to target A with a cancer-selective context (input B) that can result from target density (high in cancer, low on normal cells), or physico-chemical properties of the tumor micro-environment (TME). Created with BioRender.com.

TCE and CAR-T therapies bypass the need for cognate peptide Major Histocompatibility Complex/T cell Receptor (pMHC/TCR) recognition and activate T cells by triggering TCR or CAR signaling through direct engagement of surface tumor associated antigens (TAAs) on cancer cells (**Figure 1A**). In hematological malignancies, TCEs and CAR-Ts target lineage-specific antigens expressed on normal cell types (B cells, plasma cells) that are, at least temporarily, dispensable, enabling sustained tumor exposures while maintaining an acceptable safety profile.

In contrast, it has been much more challenging to achieve meaningful clinical activity for TCE and CAR-T therapies in solid tumors, in large part because most solid tumor TAAs demonstrate some level of normal tissue expression on non-dispensable cell types, leading to on-target/off-tumor toxicities and unfavorable tolerability profiles [even when levels of expression in normal tissues are undetectable by immunohistochemistry (10)]. This major limitation is exemplified by TCEs targeting CD70 (10), CDH3 (11) (clinical trial ID number NCT02748837), CLDN18.2 (12) (NCT04260191), EGFR (13), EPCAM (14), GUCY2C (15) (NCT04171141), MSLN (16), MUC12 (17), PSMA (18) (NCT04822298 and NCT04740034) that were all discontinued after showing evidence of on-target normal tissue inflammation in non-human primates or demonstrated unacceptable safety profiles in Phase 1 clinical trials; notably, these programs all used binders that had high affinity for their respective targets, a feature that may have contributed to their unacceptable therapeutic index. A similar high failure rate has been observed with CAR-T cell therapies targeting solid tumor TAAs, with some failures clearly attributable to on-target normal tissue toxicity (MART1, CEA, CA9, HER2) (19), including severe pulmonary toxicity from a MSLN-directed CAR-T (NCT03054298) (20). Notably, even though a TCE against CLDN18.2 was discontinued (12), and a TCE against GPC3 demonstrated a relatively narrow therapeutic index and modest efficacy (21), CAR-T clinical candidates targeting CLDN18.2 (satri-cel/CT041, trial number CT041-CG4006) (22) and GPC3 (C-CAR031, NCT05155189) (23) demonstrated promising activity/safety profiles in phase I clinical testing, highlighting that in some instances, TAAs may be unsafe to target with a TCE, but not with a CAR-T construct.

Therefore, the first and only TCEs approved in solid tumors target highly selective lineage markers that are minimally expressed in normal adult tissues: tebentafusp against a melanocyte-specific gp100-derived pMHC in uveal melanoma (UM) (24), and tarlatamab (25) targeting DLL3, a neuro-epithelial development marker in small cell lung carcinoma (SCLC) (26); likewise, the first approved engineered T cell therapy in solid tumors (Afamitresgene autoleucel) expresses a TCR (TCR-T therapy) that targets a highly selective MAGE-A4 derived pMHC expressed in a very limited sub-population of sarcoma patients carrying select HLA02-01 alleles (27), emphasizing that cancer-selectivity has only been achieved in rare indications and that cancer-selective TAAs for major epithelial cancers remain elusive.

In addition, while clinically meaningful and highly encouraging in late-line patients, objective response rates to approved solid tumor TCEs or TCR-Ts (5 to 40%) consist almost entirely of partial responses (24, 26, 27), and are accompanied by a high rate of cytokine release syndrome (CRS) and by immune effector cell-associated neurotoxicity syndrome (ICANs), pointing to additional barriers for these therapies in solid tumors. These barriers include relative lack of tumor selectivity leading to systemic cytokine release, and an immuno-suppressive tumor micro-environment (TME) that hinders T cell activity (28). These topics have been extensively discussed elsewhere (29–31) and this review will focus on strategies aimed at increasing the selectivity of TCE and CAR-T therapies in solid tumors by harnessing two inputs instead of one, that have been defined previously as “logic gated” approaches (32, 33); we will expand on the notion of dual-input by including contextual inputs that combine with a TAA to achieve cancer selectivity and contrast the feasibility of implementing these approaches using TCE or CAR-T modalities.

## 2 Dual input strategies

Boolean logic is a branch of mathematics that defines logic gates as integrators of binary inputs represented by 1 and 0 into a variety of outputs (34). These logic gates, as applied to targeted T cell therapies such as CAR-Ts or TCEs, have been classified into three broad categories termed ‘OR’, ‘AND’, and ‘NOT’ (32), which integrate the presence or absence of two TAAs at the surface of cancer cells (TAA-A and TAA-B inputs) into cancer-selective cytotoxic outputs (**Figure 1B**). But researchers have also exploited non-binary and continuous variables of the tumor and the tumor micro-environment (‘analog’ inputs in electronics parlance), such as TAA density, tumor-secreted proteases, immunosuppressive cytokines, low pH, low oxygen, and extracellular ATP to enhance the tumor-selectivity of targeted therapies, thereby implementing a variation of the ‘AND’ gate we term ‘Contextual AND gate’ (**Figure 1C**).

**Figure 2:**
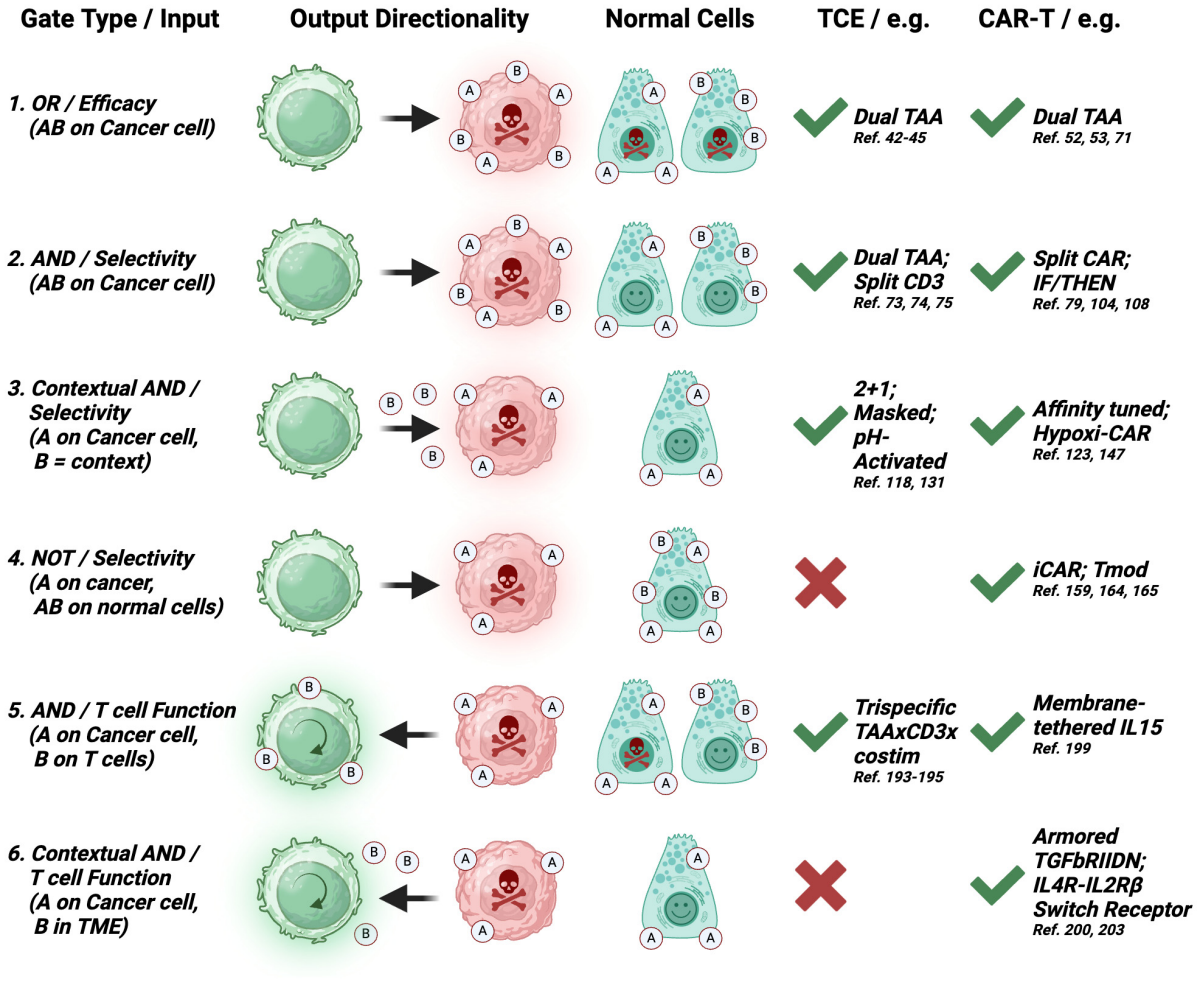
Logic gate types. Six categories of logic gates applicable to TCE or CAR-T modalities based on four types of gates (OR, AND, NOT, Contextual AND); three sources of dual-input A and B: surface target on cancer cells, surface target on T cells, TME factor; the directionality of the output: cytotoxicity against cancer cells, functionality of T cells; and the effect on normal cells expressing input A and/or B. Color code: green cells: T cells; red cells: cancer cells; red skull: cytotoxic output; green circular arrow: enhanced functionality output; green smiley: no output. The feasibility of implementation and examples for each modality are provided. Each gate type corresponds to a similarly numbered section in the manuscript. (2-1, 2-2) Both inputs are surface-expressed TAAs; (2-3) Input A is a surface-expressed TAA and input B is a tumor-selective context such as antigen density, tumor-associated proteases, low pH or low O_2_ concentration; (2-4) Input A is a surface-expressed TAA and input B is a surface-expressed protein lost only in cancer cells; (2-5) Input A is a surface-expressed TAA and input B is a T cell-expressed costimulatory receptor or CAR; (6) Input A is a surface-expressed TAA and input B is a tumor-secreted, T cell-inhibitory cytokine). Created with BioRender.com.

We further categorized dual-input strategies based on the directionality of the output (**Figure 2**) and whether it is mainly aimed at mechanistically augmenting the potency of the cytotoxic mechanism per se (e.g. an ‘OR’ gated tandem CAR-T targeting two TAAs), or aimed at increasing the number, infiltration or fitness of effector T cells, which will eventually result in better efficacy, but is mechanistically directed at the T cells (e.g. an ‘AND’ gate TCE targeting CD3 and 4-1BB). These definitions provide a more mechanistic categorization of logic gate types for each class of therapies and contrast the types of logic gates that can be engineered with CAR-T, but that are not technically achievable with current large molecules. Because of the ingenuity of the field, some designs straddle two gate types, and they will receive special mention, but we outline six main gate types based on the gate category and the mechanistic directionality of the output (**Figure 2**).

### 2.1 OR gate for enhanced cytotoxicity

For hematological malignancies, multiple approaches have been implemented to enhance clinical efficacy using “OR gate” strategies. In its simplest embodiment, an “OR gate” TCE or CAR-T therapy engages two TAAs with high potency to overcome intra- and inter-patient TAA heterogeneity, particularly problematic in AML (35, 36), or to mitigate resistance elicited by TAA loss, a common occurrence in B-ALL (37), NHL (38) and MM (39) post single-TAA therapy. A straight-forward way to deploy this strategy would be to combine two CAR-Ts or two TCEs against complementary TAAs, a promising approach currently being tested in an early trial that combines delivery of teclistamab (BCMA TCE) with talquetamab (GPCR5D TCE) in MM (40) (NCT04586426); but this strategy faces clinical development, safety and commercial hurdles that suggest it will not be broadly implementable. For example, individual TCEs go through a very rigorous dose escalation protocol to identify a tolerated administration regimen, starting at an exceedingly low and safe dose informed by the MABEL (minimally-anticipated biological effect level) approach (41); a combination of two TCEs would likely always require that a new Phase 1 dose escalation protocol be established for the combination of TCEs, something few companies who don’t own both molecules would be even willing to endeavor.

**Figure 3:**
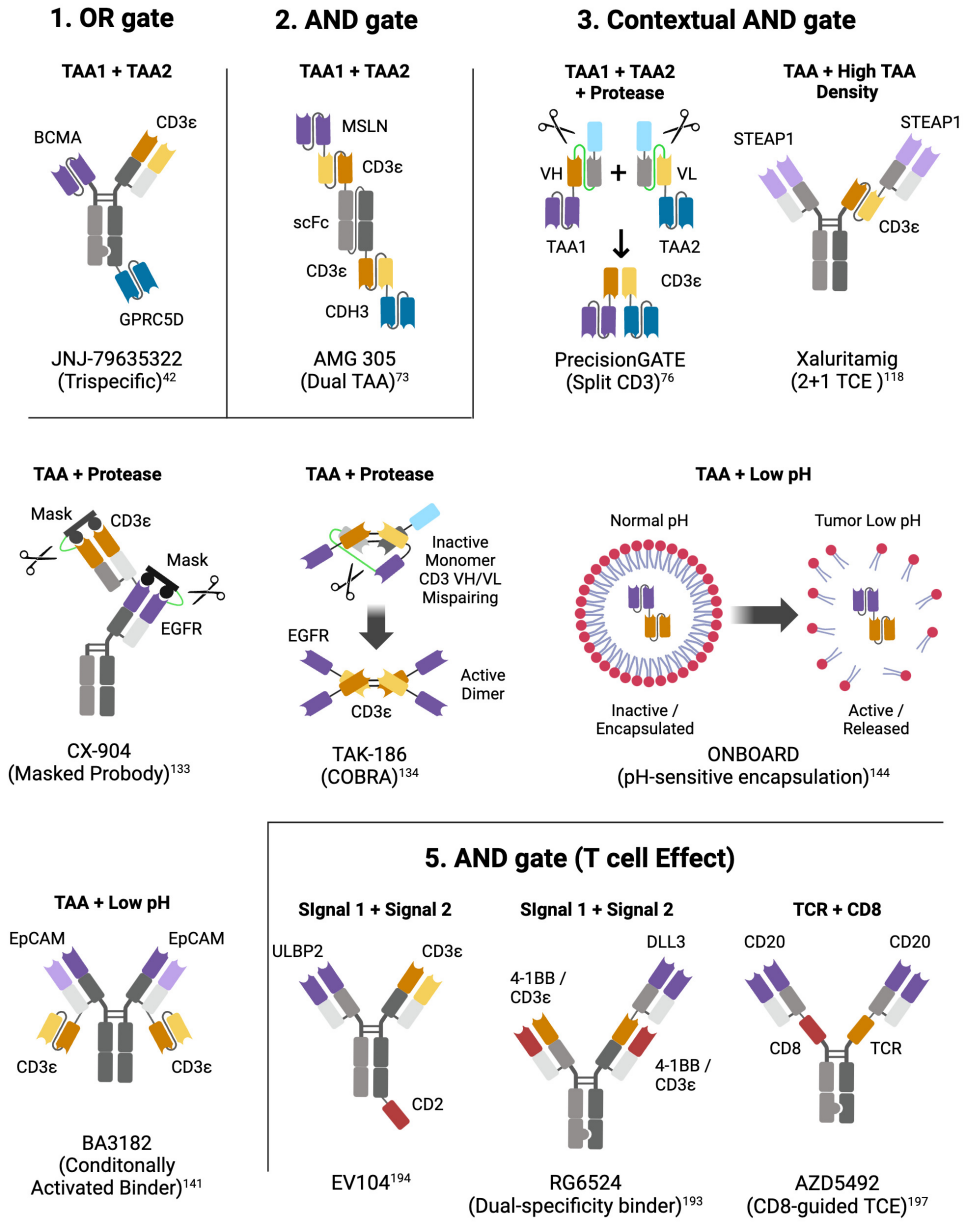
Molecular designs of select logic-gated multispecific TCEs. For each logic gate type, example(s) of multispecific TCEs are represented. Above each construct is the dual input that triggers an output (TAA, TAA density, Protease, low pH, T cell antigen); below each construct is the name of the molecule or the platform; next to each binding motif is the target of the binder; color code: purple and dark blue (TAAs), orange (CD3ε - VH), yellow (CD3ε - VL), light blue (albumin binding peptide for enhanced half-life), green (protease-cleavable linker), red (costimulatory receptor), grey (inert VH/VL, CH1/CL, CH2, CH3). Created with BioRender.com.

Rather, next generation trispecific therapies are in development that can engage either of two complementary TAAs alongside CD3, for increased efficacy or to decrease resistance through TAA loss since a tumor would have to lose both TAAs to become resistant. In MM, a BCMAxGPCR5DxCD3 trispecific OR gate TCE (42) is in clinical development (NCT05652335) (**Figure 3**-1); in B-ALL a CD19xCD22xCD3 TCE has been described (43); and in NHL, CD19xCD20xCD3 (44) (NCT05348889) and CD20xCD79bxCD3 (45) (NCT05424822) TCEs are under active clinical investigation. In AML, an OR gate TCE approach would be predicted to be challenging, since the once-robust industry pipeline of TCEs against single AML TAAs (CD123, CD33, CLL1, WT1 pMHC) has been nearly entirely discontinued (46), in large part due to high grade CRS and other adverse events at doses that produces unremarkable overall response rates (47–49). Nonetheless, a pentavalent tetraspecific (3 TAA binders, a CD3 binder and an human serum albumin binder to extend the molecule half-life) HSAxCD33xCD123xCD70xCD3 DARPin OR gate TCE (MP0533) has been described that overcomes TAA heterogeneity and improves selectivity by preferentially killing leukemic cells expressing any pair of the three targeted TAAs (50) and shows signs of clinical activity (51) (NCT05673057). Multispecific antibodies and protein constructs such as MP053, however, are very complex biologics to manufacture, as each additional binding moiety increases the risk of failure as they each need to meet exacting quality, manufacturing, stability, and immunogenicity standards and their safety profile becomes unpredictable, as they compound the on-target/off-tumor toxicity risks of each target, especially in solid tumors.

In some regards constructing a logic gate in the context of cell therapy is simpler than with a T cell engager, as the cell itself is the foundation on which to build, rather than having a soluble construct that needs to find its way to the effector cell in the body. This enables manipulation of how the cell interacts with its environment, not only controlling which cells it can effectively target, but in more sophisticated systems, enabling contextual regulation of gene expression.

**Figure 4:**
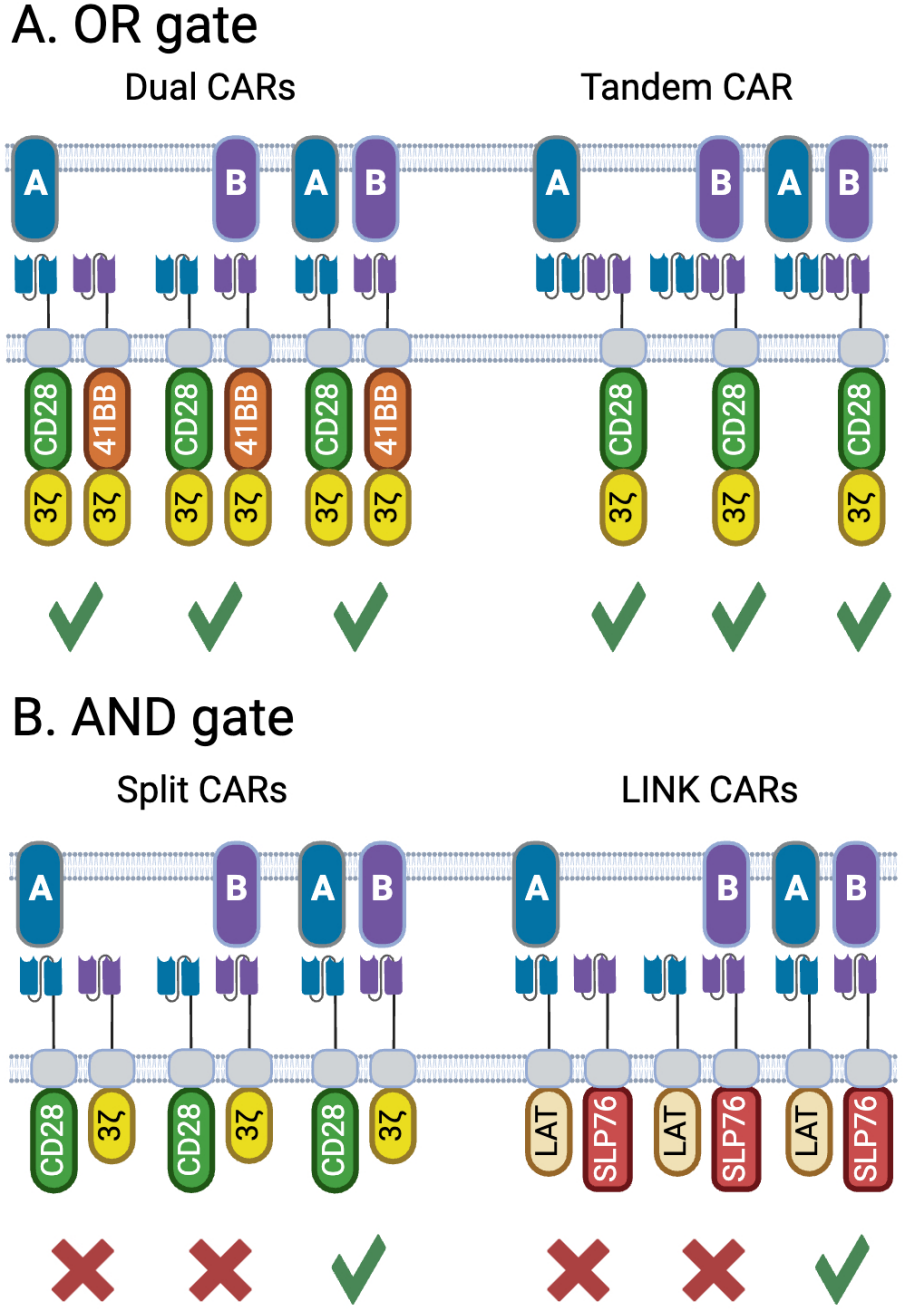
Architectures for OR and AND-gate bispecific CARs. **(A)** OR gates can respond to both TAAs either singly or together, offering an opportunity to restrict antigen escape. Two variations are shown; dual CARs which can be encoded from the same bicistronic vector, or delivered by two separate vectors, allow the use of complementary costimulation domains as shown here. Tandem CARs contain binding domains targeting two distinct antigens within the same CAR. **(B)** A true AND gate is only activated when both targets are present, and do not respond to cells expressing only one target. The split CAR concept places the domains that drive signal 1 (activation via the CD3z ITAM) and signal 2 (costimulation, shown here with the CD28 ITAM) on separate CARs. A more recent, and potentially more tightly controlled format are the LINK CARs, which place two effectors that lie downstream of the TCR (LAT and SLP76), on two distinct CARs, thus splitting signal 1 in two and requiring engagement of both CARs to initiate T-cell activation. Created with BioRender.com.

OR gates designs incorporate two or more binders into the same CAR (52–54) or two or more mono-specific CARs into the same vector (55–58) enabling targeting of cells expressing any combination of TAAs the CARs are designed against (**Figure 4A**), whereby each component CAR can be optimized separately before incorporation into the vector. Bicistronic CARs also offer the potential to use different costimulation domains on each CAR, thus gaining a broader spectrum of stimulation signals to the T cell (59, 60). Choice of targets is important to ensure that binding to one target does not compromise binding to the other, which is particularly important for tandem CARs (53). Dual or triple targeting in B cell lymphoma has been widely explored with CAR-T (61, 62), as antigen loss is a recognized cause of relapse (63), yielding insights into how to design complex multi-targeting constructs. As B cell aplasia is generally considered a clinically manageable state, targeting two or more targets that are B cell lineage markers such as CD19, CD20 and CD22, has been a common strategy for applying OR gates (64, 65). Tian at al (66). developed a bicistronic CAR construct targeting GPC2 and B7H3 for use in neuroblastoma. Both targets are over-expressed in neuroblastoma relative to normal tissue, though with a high degree of heterogeneity within tumors. Using a CITE-Seq based profiling method, CARs were identified against each target that drove optimal expansion and phenotype in the presence of target. Bicistronic CAR-Ts were able to kill both single and dual target-positive cells *in vitro* and *in vivo*, with bicistronic CAR-Ts being able to clear mixed xenografts established with NALM6 cells engineered to express each target either individually or together. In this case, the bicistronic CAR-Ts showed increased persistence with reduced exhaustion relative to the mono-CAR-Ts.

Several studies have generated CAR-T cells capable of producing bispecific T-cell engagers that can target a second antigen, thereby creating a localized OR gate CAR-T construct (67). The rationale behind this approach is to deliver a potent molecule to the tumor that targets a highly expressed tumor antigen, which may also be present on healthy tissues, thereby mitigating potential systemic toxicity. Additionally, this strategy can recruit and harness non-engineered tumor-infiltrating lymphocytes, broadening the scope of the therapeutic response.

An alternate approach using soluble adaptors can enable a common cell product to be directed to any target of choice via an exogenously administered soluble adaptor, forming the basis of the universal CAR concept. This switch module has two arms: one binds to a signaling module that resembles a standard CAR on the engineered T-cell, while the other binds to a tumor-specific antigen. While this is conceptually very similar to a T-cell engager, the Universal CAR concept enables the use of a cell product that can be optimized via engineering or processing to achieve greater consistency in response relative to T-cell engagers that rely on the presence of endogenous T lymphocytes (68, 69). Ultimately, this leads to the potential of using a uniform allogenic product that could be used ‘off the shelf’ in a range of tumor indications via the dosing of appropriate adapters. With a persistent cell product this also enables the sequencing of adapters with different TAA specificities to circumvent antigen loss, effectively serving as an OR-gate over time. Numerous Universal CAR formats have been developed and are reviewed in depth elsewhere (70). As with all T-cell based immunotherapies, universal CAR development needs to consider synapse formation; as the CAR construct on the cell product has a fixed architecture, the choice of adaptor molecule, as well as the position of the target epitope, becomes critical. It is also necessary to consider the manufacturability and pharmacokinetic properties of the adaptor (69, 70). While having one cell product simplifies targeting multiple tumors, each adaptor molecule is effectively a new drug.

However, despite recent advances in glioblastoma (GBM), where intrathecal infusion of the IL13RA2/EGFRvIII dual targeting “OR gate” CAR-T is showing very encouraging signs of clinical activity (71) (NCT05168423), “OR gate” approaches are unlikely to become broadly applicable in other solid tumor types given that achieving a safe therapeutic index with a TCE or CAR-T against a single TAA is already a significant hurdle; therefore, for solid tumor indications alternative logic gated strategies are more likely to be successful and are starting to take shape.

### 2.2 AND gate for enhanced cancer selectivity

The simplest implementations of dual-input AND gates to confer cancer selectivity over normal tissues is through binding to two TAAs co-expressed on the surface of cancer cells with no overlap on normal cells (TAA1 + TAA2 input); the resulting output (cytotoxicity) is thus only obtained when both inputs are present with no activity on single TAA-expressing normal cells.

#### 2.2.1 Dual TAA TCEs

For TCEs, this preferential engagement of dual-TAA-expressing cells results from the cooperative binding of the two targeting arms through what could be termed pseudo-avidity, whereby lower affinity toward individual TAAs contributes to the effect, but works alongside other factors, such as the structural relationships between epitopes on the two TAAs and T cells to achieve optimal distance between target cells and T cells for productive synapse formation (72), as well as the binding hindrance based on the relative position and valency of the binding domains on the multi-specific molecule. The necessity to optimize these complex inter-related factors to achieve an AND gated TCE can result in intriguing molecular formats such as the tetra-valent trispecific dual-BiTE AMG 305 (73) (**Figure 3**-2) that co-targets MSLN and CDH3, two TAAs against which single-TAA TCEs proved unsuccessful due to on-target toxicity (11, 16), but now reported to have very limited co-expression in normal tissues and high co-localization in multiple solid tumors (73). Similarly, a trispecific dual-antigen targeted (DAT) TCE recognizing B7-H4 and LY6E, TAAs that are highly co-expressed in colorectal cancer, leads to preferential killing of dual-expressing cells (74), and confirms that cooperative binding on dual-expressing cells results from the interplay of binder affinity, binding arm localization on the TCE relative to each other, and TAA density on the target cells. In practice, however, AND-gated dual-TAA targeting TCEs do not abrogate cytotoxicity on single-expressing cells; rather, they shift the potency of the cytotoxic output towards dual-expressing cells by a factor of 50 to 100-fold (73, 74). Whether this differential *in vitro* activity opens a sufficient therapeutic window to avoid on-target toxicity and whether TAA heterogeneity will limit efficacy to a small subset of patients expressing very robust levels of both targets is currently under investigation in a clinical trial for AMG 305, the first dual AND gate TCE of its kind (NCT05800964). Very recently, an ‘AND gate’ trispecific TCE targeting BCMA and CD38 has been described (ISB2001) that aims to overcome resistance encountered by bispecific TCEs in MM (75). ISB2001 clearly acts as an ‘AND gate’ TCE since potency is decreased by more than 100x when one of the targets is genetically deleted. But can it truly achieve its design goal of overcoming TAA loss given that loss of either TAA affects its activity, just as what happens in the context of bispecific TCEs? Since ISB2001 is an exceedingly potent trispecific TCE, it may nonetheless present a differentiated and more efficacious clinical profile in light of its more profound anti-tumor activity in pre-clinical models and its relative imperviousness to combined soluble BCMA, CD38 and APRIL that are often co-circulating at high levels in MM patients (75).

#### 2.2.2 Split CD3 TCEs and split CAR

An alternative to targeting two TAAs that selectively define a tumor type with a single molecule containing two TAA binders, is to combine two inactive molecules that trigger the desired output only when binding together on cancer cells. This concept forms the basis of hemibody technology, whereby the VH and a VL domains of a CD3 binder are separated and located on two distinct molecules containing different TAA binders; upon binding at the surface of dual-TAA-expressing cancer cells, the two hemibodies containing the split CD3 VH and VL domains can reassemble a functional CD3 binder capable of engaging T cells (76). While proof-of-concept was demonstrated pre-clinically, major challenges remain to develop a therapeutic platform based on the described technology, including the undesirable physico-chemical properties of isolated VH and VL domains, high clearance requiring daily dosing and potential off-target activity of each hemibody. These challenges may have been overcome by a company called Revitope using a conceptually similar split CD3 approach called PrecisionGATE (77) (**Figure 3**-3). Using half-life-extended hemibodies, optimized split CD3 complementary domains (78) and protease-cleavable masks for added selectivity (more about masking below), this third generation TCE technology has been applied to several dual-TAA pre-clinical programs for solid tumors. But while awaiting clinical proof-of-concept that this approach can work for TCEs, the CAR-T field has developed multiple versions of split CAR technologies that are simpler to implement in engineered live T cells.

The simplest form of split CAR consists of placing the activation and costimulation domains on different CARs, essentially separating signal 1 and signal 2 which are both required for optimal T cell activation (**Figure 4B**) (79, 80). This separation helps mitigate some of the issues associated with conventional CARs, such as on-target off-tumor toxicity and tonic signaling. While conceptually simple, it is critical to determine the optimal affinities for each binding domain, particularly as the activation domain-only construct in these systems is essentially a first-generation CAR and could potentially activate the T cell on its own (81, 82). Early studies showed the importance of using a weak binder on the activation domain, targeting the more widely expressed target, while using a high affinity binder targeting the more tumor-restricted target attached to the costimulatory component (80). These concepts were further demonstrated by van der Schans at al. who designed a dual split CD38/CD138 CAR for targeting multiple myeloma (83). While both CD38 and CD138 are expressed at elevated levels on multiple myeloma, they are also found on a variety of healthy tissues. A combination of low affinity CD138 CAR containing a stimulatory domain with a high affinity CD38 costimulatory CAR was shown to effectively differentiate between tumor and normal tissues. Using the high affinity CD38 binder enabled recognition and killing of multiple myeloma cells taken from patients previously treated with the anti-CD38 monoclonal antibody daratumumab who have decreased levels of CD38 expression. This highlights how a logic gated system can be designed to address the constraints found in a particular patient population.

These initial split CAR concepts still maintained intact machinery for driving either signal 1 or signal 2, which does not allow for a true Boolean-logic AND gate given that signal 1 alone can activate T cells. Tousley et al. looked at downstream T cell signaling mediators to split signal 1 between two CARs to form a true AND gate (84) (**Figure 4B**). Through gene knockouts it was shown that CARs depend on the same proximal network as native TCRs, including components such as LCK, LAT, ZAP70 and SLP76. Further analysis indicated that pairing LAT and SLP76 as the cytosolic domains of CARs targeting two independent antigens enabled bypassing of upstream signaling elements to form an AND gate that was dependent on the presence of both antigens for activation. The authors did show that it is imperative to consider the rest of the architecture of the CAR. The initial LAT/SLP76 AND gate showed a degree of leakiness attributed to target-independent dimerization of the two CARs. Given that both CD28 and CD8 hinges can drive homodimerization, the authors ensured that each CAR in the gate pair utilized a different hinge. Additionally, removal of cysteine residues in the CD28 transmembrane domain further reduced target-independent dimerization. Further mutations in the LAT and SLP76 domains were designed to remove binding sites for GRB2-family adaptor proteins. These adaptors associate with native LAT and SLP76 to form a scaffold on which PLCγ1 can associate, and it was hypothesized that the adaptors may drive target-independent clustering. These mutations eliminated any observable leakiness while maintaining efficacy in both targets’ presence. The extensive engineering required here speaks to the complexities of designing clean AND gates yet demonstrates that this concept is technically achievable.

While AND gates constrain T-cell activation to require the presence of both TAAs, the design of the binders is still complex. The affinity of each binder needs to enable engagement of each target across the full dynamic range of each target’s expression seen in tumors, which could be highly heterogenous, while maintaining differentiation from normal tissue (85–89). This is a particular challenge if there is a significant difference in the expression of each antigen, as sufficient copies of each component of the gate need to be engaged to enable tumor-specific T-cell activation. This may be complicated by the presence of either antigen on normal tissue, or in soluble form in the extracellular space, which can function as a sink for the cell. Additionally, loss of expression of one antigen from a tumor cell could lead to outgrowth of a clone within the tumor that has escaped the therapy.

An alternate AND gate configuration replaces the TAA binding domain with a soluble adapter that cross-links the cell therapy product with the target cell. While systemic co-administration of a conventional TCE with a CAR-T serves as an OR-gate (as either the CAR or the TCE alone can drive T-cell activation), expressing the TCE from the CAR-T product itself, particularly if TCE expression is regulated by CAR activation, forms a contextual AND gate as localized delivery of TCE in the tumor favors T-cell activation driven by both the CAR and TCE only in the tumor. This also enables utility of TCEs that may have dose-limiting toxicity when delivered systemically. Early conceptual studies demonstrated that engineered T-cells could secrete a TCE which could engage and activate both the producer cell and bystander T-cells (90, 91). Bicistronic constructs have been developed by several groups consisting of both a CAR and a TCE. Targeting a tumor-restricted target such as EGFRvIII in glioblastoma via the CAR component drives localization of the cell product to the tumor enabling localized release of a TCE targeting a more homogenously expressed, yet more broadly expressed, second target such as IL13Ra2 or EGFR (92–94). Interestingly in both these cases the TCE induced stronger T-cell activation and cytotoxicity relative to the CAR component, but also induced an exhausted effector phenotype, which could limit persistence of the T-cell product. Choi et al. elegantly demonstrated that expressing an EGFR TCE from an EGFRvIII CAR-T enhanced anti-tumor efficacy while improving safety (93). Including a human skin graft to their NSG mouse xenograft model, they showed that EGFR CAR-Ts both controlled tumor but also infiltrated the grafted human tissue. (Note that the binder used did not cross-react with murine EGFR). In contrast, and EGFRvIII CAR-T expressing an EGFR TCE cleared the tumor, while the human skin graft showed no signs of immune infiltration. Of course, there are no endogenous lymphocytes in this model that could engage with circulating TCE and drive on-target/off-tumor toxicity though these data are nevertheless encouraging and have led to this construct being tested in a phase 1 clinical study. Interim data, following intraventricular delivery of the cell product, showed responses in all three patients, though two of the three patients did eventually show tumor progression (67).

#### 2.2.3 IF/THEN: heterogeneous but cancer-specific markers as input

**Figure 5:**
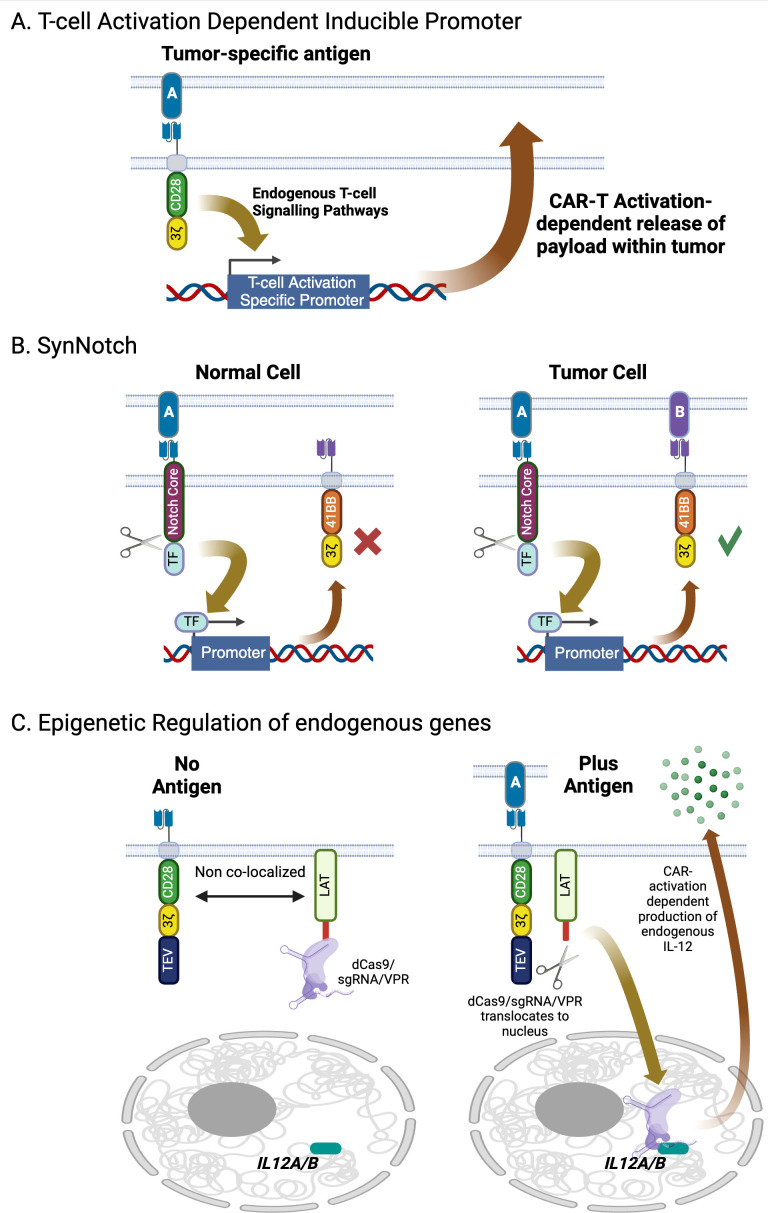
Contextual control of gene expression in engineered T-cells. **(A)** Gene expression of can be induced following CAR activation via inclusion of T-cell activation-specific promoters in the transducing vector, enabling localized delivery of immunomodulatory cytokines. **(B)** The SynNotch system offers a higher degree of specificity beyond co-opting endogenous signaling networks using an engineered transcription factor that is only released from the interior of the plasma membrane upon the SynNotch receptor engaging with its target. Payloads delivered using this system include CARs targeting antigens with significant normal tissue expression as well as immunomodulatory molecules. **(C)** The dCas9 epigenetic regulator is a highly modular system that can activate or repress expression of endogenous genes upon CAR activation. Targeted gene expression is driven by co-introduction of guide RNAs, which can be multiplexed to target multiple genes simultaneously. Created with BioRender.com.

Both OR and AND gates described thus far in the context of CAR-T cells are based on constitutively expressed components. A greater degree of control can be gained through context-specific expression of either a CAR or armament, forming an IF-THEN logic gate (**Figure 5**) (95). Conceptually, this enables a primary CAR targeting a tumor-restricted antigen that may be highly variable in its expression to activate the T cell, enabling expression of either an armament or second CAR that recognizes a more broadly expressed, but not as tightly tumor-restricted, antigen that can then eliminate the rest of the tumor while being isolated from normal tissue. While there has been significant investment in exploring regulation of CAR-Ts via application of exogenous signals such as small (96–100) and large (101, 102) molecules or physical interventions such as heat (103), these are contingent on close monitoring of the patient and are by necessity reactive to potentially debilitating conditions that will already be in progress. In contrast, contextual regulation gives greater spatial control over the CAR-T activity, in that the cells respond to their immediate environment, restricting activity to the tumor itself.

T cells already contain genes specifically expressed after activation such as PD1, CD69, NR4A, and FoxP3. The promoters for these genes contain elements that respond to T cell activation and provide a basis for designing conditional expression elements. This is the basis of the ‘TRUCK’ concept, or fourth-generation CAR where a payload, typically an immunomodulatory cytokine, is expressed downstream of a T-cell-activation dependent promoter (104, 105) (**Figure 5A**). Guo at al. successfully demonstrated repeat activation of an inducible promoter using NR4A-response elements through sequential stimulation via an anti-mesothelin CAR (106). As anti-mesothelin CAR-Ts have yet to demonstrate consistent responses in the clinic this system was designed to express pro-inflammatory cytokines in response to target engagement to boost potency. Constructs including IL2 or IL21 downstream of the NR4A promoter were shown to secrete the recombinant cytokines and increase CAR-T proliferation in response to mesothelin via CAR activation relative to controls. While minimal promoters have been successfully tested *in vitro*, it is important to remember that native genes are also regulated by an extended enhancer region. The promoter-enhancer regions can span a significant distance and cannot be packaged into a simple delivery vector. In addition, they are dependent on the overall chromatin structure that they reside in. Fraessle at al. took this into account by using CRISPR/Cas9 to directly introduce DNA encoding a CD19 CAR into the PD1 locus. Compared to control cells where the CAR had been introduced by random integration with a conventional lentiviral vector, the PD1 knock-out/CD19 CAR knock in cells responded strongly to antigen both *in vitro* and *in vivo*. Interestingly *in vivo*, absolute numbers of PD1 knock-out/CD19 CAR-T cells dropped rapidly after elimination of tumor in a xenograft model, while the constitutive CAR-Ts maintained high levels of cells in circulation. This suggests that a target-dependent feedback loop could restrain the CAR-T from moving beyond the tumor. Unfortunately, this study was unable to assess how the remaining inducible CAR-T cells could respond to tumor rechallenge due to limitations of their model. Smole at al. developed a single vector system, termed UniVect, using a constitutively expressed CAR and a payload that was inducible upon the CAR engaging with its target (107). Inducible expression was driven from an NFAT element situated upstream of a minimal promoter. This, along with the inducible payload, where placed in reverse orientation from the constitutive EF1a promoter that drove CAR expression. This is necessary to minimize interference between the two transcription elements, a significant challenge when including more than one promoter in a single vector. The UniVect system was demonstrated to successfully express IL12, a single chain derivative of the anti-IL6R antibody tocilizumab (a treatment for cytokine release syndrome) and the transcription factors TCF7 and FOXO1 which favor a more desirable memory phenotype. To address ‘leakiness’ of the NFAT promoter, endogenous TCR was knocked out of the cells via CRISPR/Cas9 to eliminate any non-CAR mediated signaling. While using T-cell activation promoters is conceptually simple, successful implementation in the clinic will require understanding how the CAR-T manufacturing process, which typically includes a CD3/CD28-based activation step, impacts and is impacted by the inclusion of these elements, leading to CAR-T products becoming activated prior to administration in patients.

The SynNotch system is based on an artificial receptor that functions in the same way as Notch, in that engagement with a specific ligand induces proteolytic cleavage of the receptor, releasing a transcription factor into the cell (108, 109) (**Figure 5B**). This transcription factor then drives an activation-specific program of gene expression. In the case of SynNotch an ectopic gene downstream of the appropriate promoter is co-introduced into the same cell as the SynNotch receptor. Such sensory networks enable the engineered cell to respond in defined ways to its environment. A striking example of this system has been designed to target glioblastoma. An anti-EGFRvIII CAR serves as the trigger. EGFRvIII is a splice variant of EGFR caused by an in-frame deletion of exons 2–7 that is tightly restricted to GBM, though shows significant intra-tumor heterogeneity (110). As such, while mono-CAR-T can effectively kill GBM cells in numerous clinical studies, in all cases EGFRvIII-negative tumor cells can escape and grow (111, 112). However, using EGFRvIII to trigger expression of a tandem CAR targeting two glioma-associated antigens, EphA2 and IL13Ra2, that are more uniformly expressed on GBM though are also found on some healthy tissues, creates a construct that significantly restricts the tumor’s ability to escape while limiting on-target/off-tumor toxicity (110). This construct has demonstrated efficacy in preclinical models and has progressed to a phase 1 clinical trial that is, of the time of writing, recruiting patients (NCT06186401). A more generalizable approach to targeting solid tumors utilizes an apelin-based SynNotch receptor (AsNR) to detect tumor endothelium (113). The constructs were shown *in vitro* to respond specifically to proliferating primary endothelial cells. Testing *in vivo* both in xenograft and spontaneous models showed that, while the engineered T cells were found throughout the mouse, only those cells located in the tumor were activated. Finally, AsNR cells were designed to produce the anti-CD19 BiTE blinatumomab in response to sprouting vessels. LLC and GL261 cells were engineered to express human CD19 and when used in xenografts were shown to be controlled by the AsNR cells expressing the BiTE, which again only activated in the tumors. It is important to note that this concept is contingent on tumors driving active angiogenesis and could have toxicity issues if a patient had active angiogenesis taking place outside the tumor, such as in a healing wound or in ischemic tissues. Nevertheless, this does demonstrate that tumor microenvironment-specific sensors could enable a more generalizable approach to tumor-specific therapeutic activation.

### 2.3 Contextual AND gate for enhanced cancer selectivity

Multiple strategies for improved cancer selectivity belong to an AND gate type that we termed “contextual AND gate” that combines the presence or absence of a TAA (binary input A) with a cancer-selective context (continuous input B, whether on cancer cells or in the TME), including cancer-selective high target density or physico-chemical TME properties (**Figure 1C**).

#### 2.3.1 TAA density/TCE and CAR-T affinity

Potentially actionable cancer-*specific* TAAs for CAR-T and TCE therapies typically result from mutations in the extra-cellular domain of transmembrane oncogenic proteins such as the recurrent EGFRvIII truncation found in up to a third of glioblastoma (GBM) patients (114), or the FGFR3^S249C^ point mutation detected in 7% of bladder cancer patients (BLCA). However, in practice, these mutations are too heterogeneous, technically too difficult to target, or too rare to be meaningfully exploited clinically. For instance an EGFRVIII-targeted TCE AMG 595 (115) and CAR-T (111) resulted in unremarkable response rates and rapid tumor adaptation through TAA loss.

A much more widespread feature of prominent and relatively homogeneously expressed TAAs is their elevated expression in cancer cells compared to normal cells; examples abound, including CEA, STEAP1, CLDN6, ENPP3, MUC16, CD70, and DLL3. Expression level is an input that can be exploited by varying the affinity and valency of TAA binders on so-called ‘2 + 1 TCEs’ to exploit avidity-mediated binding as a type of AND gate (TAA + high density). However, the affinity of 2 + 1 TCEs needs to be precisely calibrated, lest the threshold effect is obtained at the wrong target density. Cibisatamab, a CEA 2 + 1 TCE antibody, demonstrated potent killing on cancer cells expressing more than 10,000 receptors per cell and limited killing on cells below that expression threshold (116). Unfortunately, this affinity/TAA expression threshold combination proved inadequate when tested clinically, and this molecule elicited a limited overall response rate and significant on-target toxicities, likely because it was still able to engage the lower levels of CEA expressed on the basolateral side of normal enterocytes and lung epithelial cells (117). This affinity/TAA level-dependent AND gate effect appears to have been more successfully implemented in the STEAP1xCD3 XmAb 2 + 1 TCE (xaluritamig) (118) (**Figure 3**-3), which demonstrated promising anti-tumor activity (41% ORR in high dose group) in a phase 1 clinical trial associated with a manageable safety profile (119) (NCT04221542), thus opening the path for additional 2 + 1 TCEs against other differentially-expressed targets [CLDN6 (120), ENPP3 (121)] that proved too toxic when targeted with high affinity 1 + 1 TCE constructs. Given its highly differential expression profile, CD70 could be a successful candidate for a 2 + 1 TCE approach in renal cancer even though a 1 + 1 TCE approach proved too toxic (10).

The optimal binding affinity for a chimeric antigen receptor (CAR) is dependent on the difference in expression levels of the TAA between tumor and normal tissue. The affinity should allow for activation in the appropriate locations. Distinction between high and low target expression can be achieved through lower affinity binders, with potential for added control by providing exogenous small molecules to amplify the avidity of such constructs (89). Studies have shown that CAR-T cells have different activation thresholds for cytokine release, proliferation, and cytotoxicity, which must be considered when determining binder affinity (122). Increasing affinity will eventually lead to maximal T cell activation but reduced ability to discriminate tumor from normal cells. Tuning the binder affinity has been demonstrated to increase the differential activity between tumor and normal tissue for targets like EGFR (123) and ErbB3 (88). Lower affinities have the potential to separate therapeutic potency from toxicity (124). However, this is contingent on all tumor cells having higher target expression than normal tissue, which in many cases in unlikely due to heterogenous TAA expression in individual tumors. The SynNotch platform has been used to address this, using a trigger based on a low affinity binder to Her2, which depends on high levels of target expression for activation (125). This then induces expression of a high-affinity anti-Her2 CAR that can drive killing of target cells even if they display low levels of Her2. Systemic delivery of a T-cell expressing this high affinity anti-Her2 CAR would be expected to be highly toxic, but the SynNotch sensor effectively allows localized activation of the therapy within the tumor.

#### 2.3.2 Tumor protease secretion/masked TCEs

Another property of the TME that has been harnessed in recent years to attempt a more selective engagement of solid tumors is the presence of tumor-selective proteases that are inactive or not expressed in normal tissues, including matriptase, urokinase-type plasminogen activator (uPA), legumain, and MMP2/9 (126, 127). These proteases are normally produced during wound-healing or inflammation but in tumors, they are secreted and activated by proliferative or invasive malignant cells, activated fibroblasts, macrophages, or endothelial cells as a hallmark of the constantly remodeling TME (128). By constructing pro-TCEs that contain peptide masks tethered to the TAA binding site through cancer protease-cleavable linkers, several companies are testing masked TCEs against EGFR, HER2 and PSMA, TAAs that have not been amenable to safe TCE targeting in the past due to off-target or CRS toxicity (129). The field has implemented a series of improvements to solve some of the perceived weaknesses of earlier designs and was thoroughly reviewed elsewhere (130). For EGFR conditional TCEs (131–133), dual-masking was deployed to mask the TAA as well as the CD3 binder, such as in the Probody CX-904 molecule (132), due to persistent on-target toxicity concerns with single-mask approaches, whereas an elegant design called COBRA physically disassembled the CD3 binder in its inactive form to prevent any off-tumor binding (**Figure 3**-3) (134). Most masked TCEs [Pro-TriTAC (135), COBRA (134), PrecisionGATE (77), TRACTr (132, 136) and X-PAT (131)] generate a short half-life unmasked TCE post-cleavage, to ensure fast clearance and limit exposure inside tumors (130).

Interim phase 1 trial results have recently been shared that support clinical proof-of-concept that this approach can produce anti-tumor responses in patients with limited toxicity, as was intended. A trial of the PSMA XPAT molecule (JANX007) produced PSA50 responses in 5/6 patients dosed at 0.2 mg or above, but no partial response with doses up to 3 mg (NCT05519449); the EGFR XPAT molecule (JANX008) produced 1 partial response (PR) in an NSCLC patient at a 0.15 mg dose, but no additional response in 10 other patients dosed as high as 1.25 mg (NCT05783622); the EGFR Probody (CX-904) generated 2/6 PRs in pancreatic cancer patients, but no objective response in 20 other patients in the trial, across multiple indications and at similar or higher doses (NCT05387265). These results are encouraging, as they prove that the TCE MOA can produce profound partial responses in NSCLC and PDAC, and that masked TCEs can lead to clinically meaningful outcomes; but they also reveal a relative lack of dose response and a lower response rate than would be expected if unmasking was as robust in human tumors as in pre-clinical models. Ultimately, a predictive biomarker of response may be needed to stratify patients that are most likely to benefit from masked TCEs, unless the right protease-labile linker in an indication expressing robust levels of the desired protease(s) matched with the right TAA ends up producing high response rates, as may be the case for JANX007 in prostate cancer and CX-904 in pancreatic cancer.

While the masking approach has been explored extensively with large molecules, there has been little activity with masked CARs. An initial preclinical study utilizing a masked anti-EGFR CAR demonstrated the feasibility of this masking concept in the context of CARs, although it has not yet been extended to target other antigens (137).

#### 2.3.3 Hypoxia, low pH, and high ATP in the TME

The TME is characterized by unique physico-chemical properties that result from intrinsic and inter-related features of cancer cell growth and metabolism including low oxygen concentration, an acidic pH, and high ATP levels (138). Antibodies have been engineered for enhanced cancer selectivity demonstrating preferential binding at low pH (139) or high ATP concentration (140). A company called BioAtla has recently leveraged 4- to 5-fold affinity improvement at low pH for EPCAM and CD3 binders (engineered through addition of histidine switches at the CDR/antigen interface) to generate a dual Conditionally Activated Bispecific (CAB) TCE against EPCAM (**Figure 3**) with a 10-fold *in vitro* killing window between pH 6.5 versus 7.4 (141). In cynomolgus monkey, the dual CAB was tolerated at a 100x higher dose compared to an EpCAM TCE with no preferential pH binding (142). Whether the technology opens a sufficient therapeutic index (TI) for a broadly expressed TAA such as EpCAM is now under investigation in a Phase 1 trial (NCT05808634), but cell binding affinity improvements of up to 25-fold have been reported for HER2 binders at low pH (143), potentially enabling even broader TI in future pH-sensitive TCE designs. Another approach to take advantage of the low pH of the cancer TME is to encapsulate an otherwise toxic payload, such as a non-cancer selective TCE, into a pH-sensitive nanoparticle (144); this ON-BOARD platform (**Figure 3**-3) developed by OncoNano Medicine has shown proof-of-concept data in pre-clinical models with tumor-selective accumulation of the nanoparticle-encapsulated TCE in tumor lesions and anti-tumor efficacy without the accompanying weight-loss toxicity observed with the non-encapsulated payload (145). Such an approach remains clinically untested, however, and faces challenges ranging from the developability of a stable product, dosing frequency in the face of a short serum half-life, and efficient biodistribution in solid tumors.

Use of T-cell activation-specific inducible promoters has already been described above. The same principle can be used to respond to the tumor microenvironment. Cells respond to hypoxia via stabilization of the Hypoxia-Inducible Factor (HIF), a heterodimeric transcription factor. HIF contains two degradation domains that serve as substrates for oxygen-dependent prolyl hydroxylases. In oxygenated conditions HIF is hydroxylated, resulting in ubiquitylation and degradation. Under hypoxia HIF is not degraded, leading to hypoxia-dependent gene expression (146). The degradation domain has been incorporated into CARs leading to stabilization and increased expression in hypoxic versus normoxic conditions (147, 148). However, this control did come at the expense of activity relative to an unmodified CAR. Activity of the modified CARs could be restored through adding an extra layer of control using a HIF-Response Element (HRE) in the upstream promoter, leading to comparable activity to an unmodified CAR in a xenograft model. HREs have been widely explored, with numerous proofs of concept showing that using HRE promoters enables hypoxia-specific payload expression (149, 150). As CAR expression is no longer constitutive and is dependent on the CAR-T being within the tumor, hypoxia-responsive CAR-T cells exhibit reduced exhaustion and greater efficacy, with the cells exhibiting a resting state when in normoxic conditions (151). Hypoxia-regulated expression rescued a pan-ErbB CAR that was toxic in mice when expressed from a constitutive promoter. Using an optimized hypoxia-sensing promoter enabled a ‘Hypoxi-CAR’ that maintained anti-tumor efficacy while only being fully active within the tumor (152). This concept has been extended using synthetic promoters consisting of multiple components, such as response elements to IFNγ, TNFα and hypoxia, upstream of a minimal promoter (153, 154). A HER2 CAR cloned downstream of this synthetic response element was shown both *in vitro* and *in vivo* to be active in the presence of the three required stimuli. The authors chose to use multiple stimuli to gain greater specificity for the inflamed, hypoxic tumor microenvironment. Of course, a different design would be required to successfully target ‘cold’ or immune-excluded tumors. Implementation of functional high-throughput screening techniques will enable continued development and refinement of context-dependent synthetic promoters (155).

### 2.4 NOT gate for cancer selectivity

An alternative approach to driving tumor specificity is to include an ‘off switch’ which renders the therapeutic inactive in normal tissue, but which is lifted once the therapy engages the tumor. A number of approaches that utilize inclusion of domains that induce destabilization or proteolysis of the CARs have been developed where addition of a small molecule regulator blocks (156) or promotes (156) degradation of the CAR by inducing structural changes that modulate the CAR susceptibility to proteolysis. These approaches are dependent on having a small molecule regulator that has drug-like properties and that give the requisite precision of control. A similar approach has been taken with switchable bispecific T cell nanoengagers, or SiTEs, in which the CD3 and tumor antigen binding domains are not covalently linked, but instead linked via a ‘supramolecular aggregate’ enabling T-cell mediated cytotoxicity akin to a standard TCE (157). This aggregate can be broken apart by administration of the small molecule drug amantadine. However, these methods do not control therapeutic activation based on location in the tumor.

**Figure 6:**
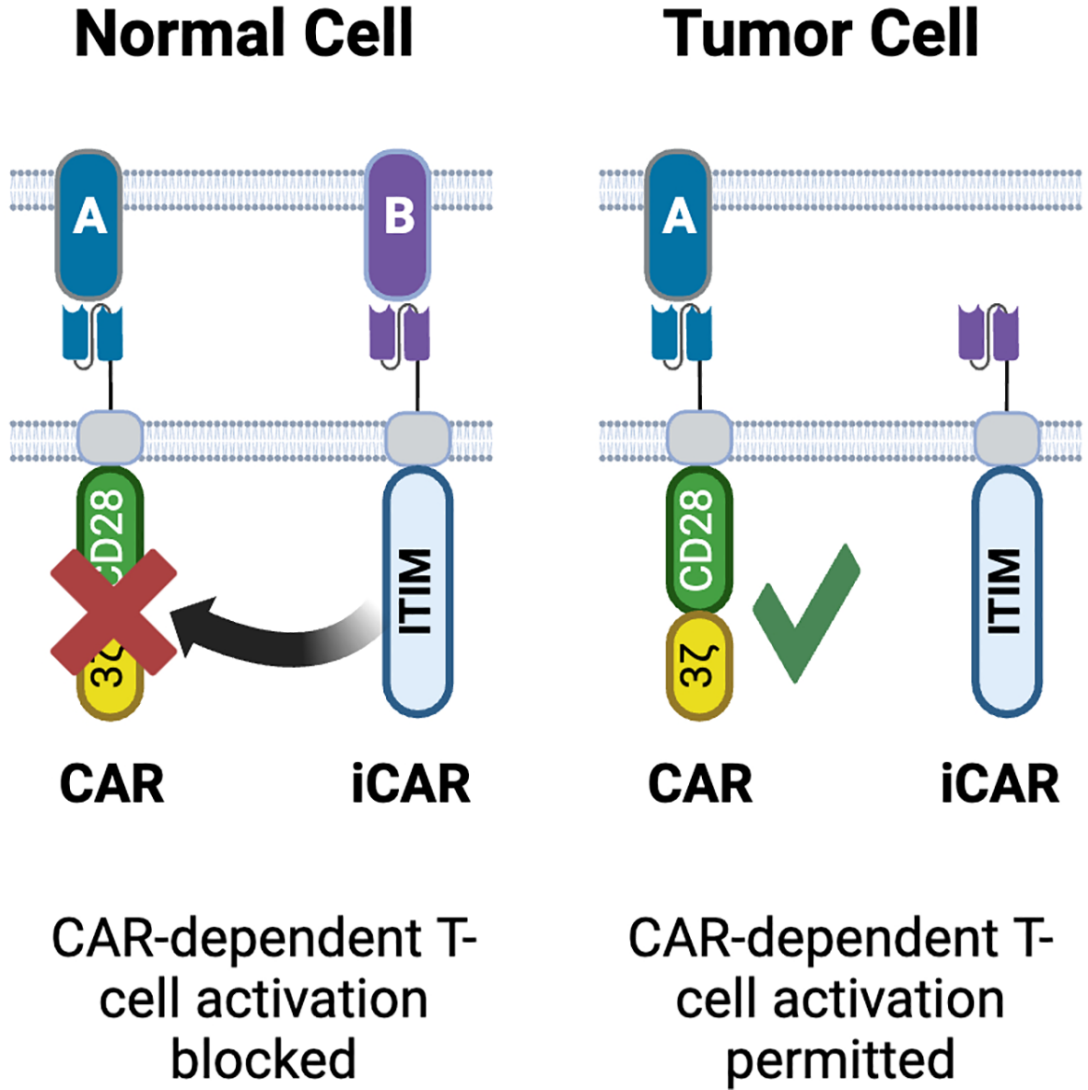
NOT gated CARs. Inhibitory CARs (iCARs) containing ITIM domains derived from PD1, CTLA4 or KIR are designed to bind targets on normal tissues but not on tumor cells. Upon engagement with its target, the iCAR blocks activation of the conventional CAR co-expressed in the same T-cell. As the iCAR target is only on normal cells, CAR-T activation occurs only in tumors. Created with BioRender.com.

The concept of NOT gate is based on differences in input localization between normal tissues and tumors and requires a target that is present on normal cells, but absent on tumor cells (**Figure 2**-4). This principle is similar to the mechanism by which NK cells are blocked from attacking ‘self’ via inhibitory KIR receptors (158). An early approach used inhibitory CARs (iCAR), which include an ITIM domain in their cytosolic region (**Figure 6**). On engagement with its cognate antigen, the iCAR produces a signal that blocks activation of the T-cell. The original implementation of this concept utilized PD1 and CTLA4 ICDs, which inhibited T cell activation while the iCAR was engaged with its target but released their inhibition when the iCAR disengaged (159). Inhibition via this NOT gate was reversible, and kinetically occurred with the T cell was in contact with the incorrect cell. These are both significant advantages over the ‘kill switch’ approach to controlling CART-mediated toxicity as the kill switch is irreversible and does not occur instantly, as symptoms first need to be observed by the physician, then the exogenous effector required for the kill switch has to be dosed, followed by a period of time where the effector can reach all the CART cells in the patient (101).

Subsequent efforts to design optimal NOT gates have identified key parameters for consideration. While Fedorov showed that kinetically there was no delay in inhibition (159), this may well have been a serendipitous discovery arising from the components chosen in their proof of concept. Bangayan at al. developed a NOT gate using a TROP2 iCAR to block activation of a CEACAM5 CAR (160). Their initial iterations failed to completely inhibit activation. Increasing the affinity of the iCAR binder failed to improve inhibition, though increasing avidity of the iCAR relative to the CAR, through modulating expression of either the iCAR or TROP2, did increase the efficiency of inhibition. This is akin to observations regarding the importance of avidity in the design of conventional CARs, and for a NOT gate to function effectively, careful quantification of both the CAR and iCAR, as well as the expected quantities of targets in the patient, needs to occur. Funk at al. also observed a delay in iCAR inhibition that failed to prevent on-target, off tumor toxicity (161). Interestingly although the iCAR in this study was unable to block cytotoxicity, it was able to block T cell proliferation and cytokine production. The authors speculate that this is indicative of inhibition of *de novo* transcription and translation (required for proliferation and cytokine production), while cytotoxicity is reliant on release of existing proteins such as granzyme and perforin that are stored in granules.

HLA (human leukocyte antigen) loss of heterozygosity (LOH) offers a practical approach to using the NOT gate concept in a clinical context (162). LOH arises from the loss of large regions of DNA in tumor cells (on average around 20% of the genome is affected per tumor cell). NGS has revealed that LOH is frequently clonal, arising early in tumor evolution leading to a high degree of homogeneity in LOH within a given tumor. The Tmod platform takes advantage of this phenomenon, with a modular system that is based on HLA-specific single chain antibody variable region fragments (scFv) as their iCAR binding domain fused to the hinge, transmembrane and cytosolic domains of Leukocyte Ig-like receptor (LIR)-1 (163). LIR-1 serves as an inhibitory receptor the recognizes self via interactions with MHC class 1. The Tmod blocker domain is paired with a conventional tumor antigen targeting CAR. *In vitro* and *in vivo* proofs of concept have been achieved with both mesothelin (MSLN) (164) and carcinoembryonic antigen (CEA) (165) targeting Tmod cells. In both cases the Tmod blocker is based on an scFv that specifically recognizes HLA A*02. Both targets, despite showing enriched tumor expression, have multiple cases of dose-limited toxicity arising from on-target/off-tumor activity (116, 166–168), making them ideal targets for the Tmod platform. The development of both the MSLN- and CEA-Tmod followed the same process, speaking to the modularity of this platform. A key feature of the Tmod design process is to gain a quantitative understanding of the relative expressions of tumor target and HLA A*02 across normal tissues and patient tumor samples (164). This enables CARs with an EC50 (based on target copy number) that exceeds the expression of target on normal tissues. For CEA, many solid tumor indications, including colorectal, pancreatic and lung, expressed CEA above the CAR EC50, though normal colon and esophagus also expressed elevated levels of CEA. Fortunately, these tissues also expressed HLA A*02 well above the IC50 of the Tmod blocker. Use of both mixed and serial cultures showed that the Tmod constructs could not only distinguish between tumor and normal cells (defined as both cells being CAR TAA-positive, with the tumor cells being HLA A*02-negative and the normal cells being HLA A*02-positive) in a mixed culture, but also showing that the kinetics of the Tmod system were fast enough to enable the Tmod blocker to inhibit CAR-T cell activation when the Tmod cells were transferred from a tumor to a normal cell culture, with no cytotoxicity being observed in the normal cells. Conversely transferring Tmod cells from coculture with normal cells, where no cytotoxicity is observed, to coculture with tumor cells resulted in tumor cell killing. *In vivo* both MSLN-Tmod and CEA-Tmod cells could distinguish between HLA A*02 positive and negative tumors that were implanted on opposing flanks of the mice. These data suggest that control of CAR-T activation and inhibition is sufficiently tight as to prevent on target/off tumor activity. It is important to note that cis-binding of the Tmod blocker to HLA A*02 on the surface of the engineered T-cell can compromise activity; this was observed both in transgenic Jurkat cells over-expressing A*02 and in primary T-cells. Inclusion of an shRNA to reduce β2-microglobulin expression in the T-cell reduced surface expression of A*02 and restored Tmod functionality. Both programs have progressed to phase 1 clinical trials (169–171) (NCT06051695, NCT05736731), which are paired with a pre-treatment diagnostic test to confirm HLA type and LOH of the HLA locus in the tumor (172). It is recognized that mediating control via HLA recognition does limit the treatable population to those patients with that HLA type, with the A*02 haplotype being prevalent in Caucasian populations (173). The Tmod platform has been shown to be highly modular and can function with other HLA haplotypes such as A*11, the most prevalent class I allele in Asian populations (174). Additionally it is postulated that the proportion of patients whose tumors exhibit LOH at the HLA locus will increase with the growing use of immune checkpoint inhibitors (ICI), as LOH is a recognized negative predictor of overall survival in ICI patients (175).

While highly complex, NOT gates are theoretically implementable with trispecific TCEs using dual-specificity binders: for instance, a TAAxβ2MxCD3 TCE carrying a well-designed dual-specificity β2M/CD3 binder (high affinity for β2M and low affinity for CD3) could prevent CD3 engagement against TAA+/β2M+ normal cells, while permitting engagement on TAA+/β2M- cancer cells [given that a substantial fraction of solid tumors lose β2 microglobulin via genetic or epigenetic mechanisms to evade anti-tumor immunity (176)]. In practice, however, since β2M is expressed by all normal cells in the body, such a molecule, even if it were achievable to engineer, would likely suffer from unsurmountable target-mediated drug disposition (TMDD) and would likely be cleared before reaching the tumor site. Since tissue-restricted plasma-membrane proteins that are recurrently lost by cancer cells have not been identified to date, NOT gate approaches will likely only be implementable with elegant CAR-T designs.

### 2.5 AND gate and contextual AND gate for enhanced T cell function

Multiple studies have demonstrated improved anti-tumor activity of TCEs in the presence of a T cell costimulatory signal; whether in combination with a 4-1BB agonist (177) or a targeted CD28 bispecific antibody (178), or when CD58 is present at the surface of cancer cells to stimulate CD2 T cell signaling (179). These studies build on an extensive body of work from the CAR-T field establishing the crucial contribution of so-called signal 2 (costimulatory signal) (180) to the survival, proliferation and efficacy of engineered T cells *in vivo*. As a result, antibody engineers have recently developed a new generation of trispecific TCEs that incorporate a costimulatory domain to improve the proliferation of T cells recruited by TCEs and counter the dysfunction phenotype that results from repeated TCR stimulation (181), thereby generating AND gate effects functionally directed at the T cells (**Figure 2**-5).

Researchers at Zymeworks have developed a DLL3xCD3xCD28 trispecific TCE (TriTCE) with improved anti-tumor activity and increased T cell activation, proliferation and cytokine production (182). They were preceded by a team at Sanofi who described a TAAxCD3xCD28 trispecific TCE platform that was applied to the CD38 (183) and HER2 (184) TAAs and also demonstrated increased potency, T cell activation and cytokine release compared to bispecific controls. While some of these T cell enhancement features are desirable, such a design with high affinity (single digit nM) binders for all three binders in the trispecific TCE ran the risk of enhanced toxicity like CRS or on-target/off-tumor activity, and both assets were discontinued after phase 1 clinical trial initiation (NCT04401020, NCT05013554). One would conclude that a measure of the likely success of a trispecific costimulation-enabled TCE platform would be enhancement of T cell health (long-term killing potential, proliferation) with the same or preferably lower cytokine release compared to a benchmark bispecific TCE, likely necessitating adjustments to the relative affinity of the CD3 and costimulatory binders. Simultaneous engagement of CD3 and a costimulatory receptor with a trispecific TCE could preserve long-term T cell functionality by preventing activation-induced T cell death (AICD) (185) and T cell anergy (186) that are exacerbated with TCE signaling absent costimulation. Affinity and off-rate optimization have been successfully achieved in the past to decouple T cell killing function from cytokine release (187–190), leading to therapeutics with potentially superior efficacy/toxicity profiles compared to higher affinity CD3 TCEs (191, 192).

With such design goals in mind, Chugai has developed a unique DLL3xCD3x4-1BB trispecific TCE (**Figure 3**–5) that contains two dual-specificity CD3/4-1BB Fab domains that can stimulate CD3 and 4-1BB reporters with a comparable EC_50_ and a much reduced CD3 affinity (K_D_ ~ 1.5 μM) (193). With the same objective, a team at EvolveImmune engineered the elegant Evolve platform consisting of trispecific TAAxCD3xCD2 TCEs containing an affinity-optimized CD3 binder and the engineered extra-cellular domain (ECD) of CD58 to costimulate the CD2 T cell co-receptor (**Figure 3**-5), leading to greatly enhanced T cell proliferation upon repeat-stimulation assays but with moderate accompanying cytokine release (194). A comparable design was unveiled by Novartis for a CD19xCD3xCD2 TCE (PIT565) also containing a CD58 engineered ECD (195); however, an emphasis on enhanced potency and cytokine production compared to bispecific controls suggests they may not have incorporated decreased cytokine release relative to target killing into their design; a future update on their clinical trial testing (NCT05397496) will reveal if the activity/safety profile of PIT565 will prove superior to other next-generation CD19 TCEs currently in clinical development such as AZD0486 (NCT04594642), a TCE developed to limit cytokine release using a differentiated CD3 binder (196).

Finally, another AND gate design (directed at T cell functionality) called CD8-guided TCE (**Figure 3**) was recently unveiled by AstraZeneca consisting of a CD20xTCRxCD8 trispecific TCE (AZD5492) designed to selectively engage CD8+ T cells and avoid CD4+ T cells, resulting in potent anti-tumor activity and a significantly decreased cytokine release profile (197).

There are numerous examples of enhancing fitness of CAR-T cells through both manipulating manufacturing conditions (198) and through constitutive expression of both positive regulators of T cell activation such as membrane-tethered IL15 (199) or dominant negative regulators to block inhibitory signals, such as TGFβ (200) from the tumor microenvironment. This category of logic-gated CAR, called armored CAR-T, exemplified in **Figure 2**-5 includes dominant-negative receptors that can inhibit PD-L1 (201) and FAS-L (202) signaling in CAR-T cells, or chimeric “switch-receptors” that bind an immune-suppressive cytokine such as IL-4 and GM-CSF and turn it into a positive signal 3 for T cells via, respectively, IL-2Rβ (203) or IL-18R (204) signaling. However, some of the most potent stimulators of T cell function such as IL2 and IL12 can induce systemic toxicities and could lead to target-independent proliferation if expressed constitutively from the cell product. SynNotch receptors have been used to drive target dependent expression of IL12 from NK cells (205, 206) and IL2 from CAR-T cells (207). In the latter case, IL2 produced by the tumor-responsive SynNotch circuit drove autocrine stimulation of the T-cell in a tumor specific, but TCR/CAR independent, manner enabling efficient tumor infiltration and control of an immune-excluded tumor model.

These systems require a recombinant transgene to be delivered to the cell to serve as the payload that is triggered by the SynNotch receptor. An alternative approach using an engineered catalytically dead version of Cas9 has enabled epigenetic control of endogenous genes in response to tumor markers (**Figure 5C**) (208). This bipartite system includes a second-generation CAR that has a TEV protease domain added to its C-terminus and LAT (linker for activation of T-cells) fused to a nuclease-deactivated dCas9 and the transcriptional activator domain VPR. Upon target engagement by the CAR, the two components come into proximity as part of the immune synapse. The protease cleaves the dCas9-VPR fusion from the LAT peptide, allowing the former fusion protein to move away from the plasma membrane. Co-introduction of appropriate guide RNAs enables the dCas9 domain to bring the VPR transcription activation domains to the promoter of the gene or genes that are to be activated. This was successfully demonstrated using an anti-HER2 CAR as the trigger, and guide RNAs to promote expression of two endogenous genes, IL12A and IL12B, that encode the p35 and p40 subunits of IL12 (209). Activation is dependent on presence of TAA and is switched off when the target has been eliminated. The authors also demonstrated that, while IL12 was produced from the cells at levels sufficient to improve CAR-T activity, it was still low enough to not lead to detectable systemic IL12. It is also conceivable that a greater number of genes could be regulated in this fashion by introducing additional guide RNAs into the system. It is important to note that manufacture of cells with the dCas9 epigenetic regulator requires transduction with two lentiviral vectors given the size of the constructs required, which adds additional costs and complexities to the manufacturing process. This can be addressed either through non-viral gene delivery systems that have larger capacities than the lenti- and retroviruses used in all approved cell therapies, or via protein engineering to reduce the size of the components. An engineered variant, CasMINI, has approximately 500 amino acids, compared to 1000-1500 amino acids found across the Cas9 and Cas12 families, and has demonstrated robust gene and base editing and, in its nuclease dead form, gene activation activities (210, 211).

## 3 Discussion

In this review, we have outlined “logic gated” strategies that are in pre-clinical or clinical testing that could be applicable to solid tumor TCE or CAR-T, and we discussed the combination of key factors that will likely dictate efficacy and safety in patients: (i) TAAs, (ii) modality, (iii) engineering.

Choosing the appropriate TAA binder(s) for the intended indication remains the most crucial factor: (i) to overcome TAA heterogeneity or loss associated with intrinsic or acquired resistance, the right “OR gate” partners need to be identified, (ii) to maximize the chance of success of an “AND gate” strategy, two TAAs with high, homogeneous, and overlapping tumor expression are required, while having no co-expression in any critical normal tissue. AMG 305 may best exemplify such a valuable “AND gate” TAA pair, anchored by MSLN, a mostly mesothelial marker, amenable to combination with the mostly epithelial CDH3 marker (73). Such TAA pairs do not abound and target discovery for these pairs is fraught with difficulties, thus it may be simplest to find a novel partner TAA to combine with a well-validated anchor TAA with known liabilities rather than rely on unbiased searches that will open a Pandora’s box of possible unvalidated TAA pairs. Rescuing a validated TAA that has shown clinical promise but normal tissue toxicities through “contextual AND gate” is the most advanced approach with multiple masked TCEs in clinical development.

While there are advantages to each modality that have been outlined elsewhere (212), a valuable lesson from the hematological malignancy space is that TCE and CAR-T therapies are complementary and offer different risk/benefits profiles that are weighed daily by clinicians, patients, and payors to unlock the most beneficial outcome for each patient. Here, we have contrasted the technical merits of each modality to achieve logic gated outcomes. CAR-T have advantages and versatility whereby the T-cell provides the chassis on which logic gate systems can be built, including those requiring multiple independent components (such as SynNotch and dual-CAR), limited only by how much genetic material can be introduced into the cell. Because of this modular capability, CAR-Ts can be engineered to implement every type of logic gate, whereas TCEs are limited to what can be engineered in a single molecule and for example cannot yet implement the NOT gate (**Figure 2**). Lentiviral and retroviral vectors are widely used but have capacity constraints; moreover, including a second inducible promoter in addition to the main viral promoter is complex given the potential for promoters to interfere with each other. While considerable efforts to develop alternate gene delivery methods that do not have these constraints have been undertaken, such as transposon technology (213), these have yet to demonstrate success in the clinic. Allogenic platforms offer the potential for engineering additional complexity, particularly as base-editing technologies enable multiplexed simple engineering to knock out genes with single base changes (single strand cut rather than double strand cut from CRISPR). shRNAs could also enable modulation with a smaller effector. Harnessing endogenous gene expression via epigenetic regulation (particularly with multiplexing) permits greater opportunities for broader contextual manipulation of gene expression.

In addition, engineered CAR-T enable the implementation of more binary outputs, whereas logic gated TCEs mostly open the therapeutic index by shifting the activity of the molecule towards cancer cells. Therefore, masked TCEs still induce a certain level of CRS, and dual-TAA AND gate TCEs still engage single TAA-expressing cells (albeit at 100-fold higher concentrations, but such concentrations are often reached shortly after intra-veinous dosing). Potentially more binary TCE designs, exemplified by the very elegant COBRA (134) and PrecisionGate (77) platforms (**Figure 3**), look promising pre-clinically but remain in need of clinical validation. Moreover, truly binary outputs, while perhaps desirable, may be unnecessary if 2 + 1 TCE designs (**Figure 3**) can consistently exploit TAA expression differences between normal and tumor tissues, as shown with the STEAP1 TCE xalutiramig (118, 119) and currently in phase 1 clinical testing for a CLDN6 (120) and an ENPP3 (121) TCE. In future iterations, double AND gated approaches, whereby dual-TAA TCEs will also be outfitted with a costimulatory receptor binding domain, will likely emerge, enabling selectivity and enhanced T cell function in the same molecule.

But what if the ultimate logic-gated construct of the future was one that incorporated the best features of both modalities? One of the most discussed and exciting CAR-T design (OR gate) developed thus far is the CARv3-TEAM therapy developed for GBM, that secretes a short-lived TCE against EGFR, thereby co-engaging bystander T cells alongside the cancer-specific anti-EGFRvIII CAR T cells (67). This may represent the best use of both technologies, whereby the CAR-T therapy serves as a cancer-homing and a tumor-localized synthesis unit that cooperates with endogenous T cells engaged by a TCE it encodes and secretes. Cell therapy has shown that cures for advanced hematological cancers is achievable; engineered to deploy gated-logic outputs, and in conjunction or in parallel with more selective and potent logic-gated TCEs, they will undoubtedly be a key component of future cures in solid tumor indications.
